# National trends in loneliness and social isolation in older adults: an examination of subgroup trends over three decades in Sweden

**DOI:** 10.3389/fpubh.2024.1444990

**Published:** 2024-09-11

**Authors:** Lena Dahlberg, Isabelle von Saenger, Mahwish Naseer, Carin Lennartsson, Neda Agahi

**Affiliations:** ^1^School of Health and Welfare, Dalarna University, Falun, Sweden; ^2^Aging Research Center, Karolinska Institutet and Stockholm University, Solna, Sweden; ^3^Department of Sociology and Work Science, University of Gothenburg, Gothenburg, Sweden; ^4^The Swedish Institute for Social Research, Stockholm University, Stockholm, Sweden

**Keywords:** loneliness, social isolation, social connection, older people, trends, gender, mobility impairment, mental health

## Abstract

**Introduction:**

Loneliness and social isolation are public health concerns. This study aimed to examine levels and trends in loneliness and social isolation among older adults (77+ years) in Sweden, assess subgroup variations, and determine associations between loneliness and social isolation.

**Methods:**

The 1992, 2002, 2004, 2011, 2014 and 2021 waves of the Swedish Panel Study of Living Conditions of the Oldest Old (SWEOLD) were analysed through ordered logistic and linear regressions.

**Results:**

On average, 12.5 percent of the participants experienced loneliness often/nearly always, while 6 percent were categorised as severely isolated. Loneliness and social isolation were more common in women, those aged 85+, and persons with basic education, in psychological distress or with mobility limitations. Loneliness was also associated with living alone. No increases in loneliness or isolation were identified; instead, loneliness tended to decrease in groups already experiencing lower levels of loneliness. Loneliness and social isolation were moderately associated each survey year.

**Discussion:**

This study challenges perceptions of high and increasing levels of loneliness and social isolation. Given the impact on health and wellbeing and the ageing of populations, policy and practice should still address these issues and target vulnerable groups. Subgroup analyses in trends are scarce and should be explored further in future research.

## Introduction

Loneliness and social isolation have been neglected social determinants of health (1), even though there is substantial evidence that they increase the risk of poor physical and mental health, well-being and premature mortality (2–4). The World Health Organization (1) states that loneliness and social isolation are growing public health and public policy concerns [see also Prohaska et al. (5)] and has launched a commission on social connection aiming to make loneliness and social isolation “recognized and resourced as a global public health priority” (6). There has also been a growing research interest in loneliness and social isolation since 2020, with a peak during the COVID-19 pandemic (7), when strategies to prevent the spread of the virus severely restricted social interaction. Older adults have been identified as an age group with higher prevalences of loneliness (8) and social isolation (9). Based on a national survey, this article examines levels and trends in loneliness and social isolation in older adults over a 30-year period and assess variations across subgroups.

Loneliness and social isolation are related but distinct concepts [see, e.g., (10)], indicative of subjective and objective aspects of social relations, respectively. While loneliness is a feeling arising due to discrepancy between a person’s desired and achieved levels of social relations (11), social isolation is an objective state characterized by a lack of social contacts with markers such as living alone, having a small social network and infrequent social contacts (12). So, while social isolation is a risk factor for loneliness (13), not all socially isolated persons experience loneliness, since the experience of loneliness results from a cognitive appraisal of one’s social relations with respect to individual social standards such as perceptions and expectations (14).

### Prevalence and trends in loneliness and social isolation

A meta-analysis found that the pooled prevalence of loneliness among people aged 65+ years in high-income countries was 28.5 percent, with a higher prevalence in people aged over 75 years (31.3%) than in those up to 75 years (27.6%). The proportion experiencing severe loneliness (highest levels of intensity or frequency) was considerably lower at 7.9 percent (15). Another meta-analysis estimated the pooled prevalence of social isolation to be 25.0 percent in community-dwelling people aged 60 years or older, which may be an underestimation given that people living in institutions were excluded from the analysis (16). Yet another meta-analysis, focusing on the situation during the COVID-19 pandemic, estimated that 28.6 percent of people aged 65 years or older experienced loneliness, while 31.2 percent experienced social isolation (17). It should be noted, though, that prevalences are dependent on cutoffs defining the presence of loneliness and social isolation and that the cutoffs vary across studies.

A systematic review identified four repeated cross-sectional studies reporting multiple comparable prevalence estimates of loneliness (8), of which one study concerned older adults, showing no significant change in the trend between 1992 and 2014 in Sweden (18). Similarly, no significant change in loneliness among older adults was found over a five-year period in the Netherlands (19), a 10-year period in Sweden (20) and a 10-year period in the United states (21), while decreases in loneliness were found in 20-year follow-up studies in Finland (22), Germany (23) and the United States (24).

Regarding social isolation, trend studies are scarce. Still, a recent study in the United States identified an increase in social isolation among the general population in the last two decades, but although the levels of isolation were highest in older adults (65+), no increase in isolation was identified in this age group (9). Albeit there have been mixed findings and a general lack of nationally representative or well-stratified samples has been noted, studies generally found an increase in loneliness and social isolation in older adults during the COVID-19 pandemic (25–27), especially among studies conducted more than 3 months into the pandemic (17).

### Subgroups at risk of loneliness and social isolation

Systematic reviews have found an increased risk of loneliness related to, e.g., non-married/-partnered status, partner loss, limited social network, low level of social activity, poor self-perceived health, poor mental health, poor functional status, women, higher age, and lower educational level (13, 28).

Similar risk factors have been identified in reviews on social isolation, including physical and mental health problems, functional limitations and low education, whereas findings on age are mixed (29–32). Social factors such as not having a partner/living alone and no social participation have also been found to predict social isolation (29, 32), at the same time as such factors can be used as indicators of social isolation (33). Unlike loneliness, where women are at higher risk, men have been found to have smaller social networks than women and to be more vulnerable to social isolation (29, 32).

### Trends in subgroups of older adults

Trends in loneliness and social isolation may not be the same for all segments of the older population, and subgroup patterns can inform targeting of interventions by identifying groups particularly vulnerable to loneliness and social isolation. Although analyses of variations in trends in loneliness and social isolation across groups of older adults are rare, studies have found increases in loneliness in those with activity/mobility limitations (19) or with cognitive impairment (20). A recent study concluded that trends in loneliness were similar across most subgroups, but that the trend was a greater increase in loneliness among those born 1928–1945 (than those born 1901–1927 and 1946–1964) and in widowed participants (24). In the general population (aged 15+ years) in the United States, women were found to be more socially isolated than men, measured as hours spent with nobody else. However, while both groups experienced an increase in social isolation from 2003 to 2020, this increase was greater in in men (9).

### Aim

The aim of this study was to examine levels and trends in loneliness and social isolation among older adults in Sweden over three decades, assess variations across subgroups, and determine the strength of association between loneliness and social isolation over the years.

## Materials and methods

### Design and participants

This study has a repeated cross-sectional design. Data is drawn from the Swedish Panel Study of Living Conditions of the Oldest Old [SWEOLD (34)]. SWEOLD includes individuals aged 77 years or older living in Sweden at the time of the interview, and recruits individuals who have been randomly sampled to participate in the Swedish Level of Living Survey (LNU) and have reached the upper age limit of that study. In 2011 and 2021, additional samples were drawn to ensure the inclusion of the oldest old. SWEOLD data was collected in 1992, 2002, 2004, 2011, 2014 and 2021. The last wave of data collection occurred between June 2021 and May 2022, that is, toward the end of the COVID-19 pandemic.

### Procedure

In 1992, 2002 and 2011, the main mode of data collection was face-to-face interviews in the person’s home, while some participants were interviewed via telephone. In 2011, self-completion of postal questionnaires was also offered. In 2004, 2014 and 2021, telephone interviews were used as main data collection mode, with the option of self-completion of questionnaires. For individuals who were unable to answer questions themselves due to, for example, cognitive impairment, proxy (or mixed) interviews were conducted with a spouse/partner or another close person. Response rates ranged between 95.4 percent in 1992 and 63.9 percent in 2021. In this study, only individuals who were directly interviewed were included, with a total analytical sample of 3,487 individuals. All interviewers were trained and informed consent was obtained from each participant prior to the interview. The study has received ethical approval by the Swedish Ethical Review Authority (reg. no. 2019–06324, 2021–00393, 2022–01079-02).

### Materials

#### Dependent variables

Loneliness was measured via the single item ‘Are you often bothered by feelings of loneliness?’ [response options: almost never (0); seldom (1); often (2); nearly always (3)].

Social isolation was measured via an index comprising three indicators: living alone; lack of social contacts with children and grandchildren; and lack of social contacts with relatives and friends.

Living alone was measured via the item ‘Do you live alone?’ [yes (1); no (0)]. Participants living in care homes were regarded as living alone.

Lack of social contacts with children and grandchildren was based on two items on frequency of contacts with children and with grandchildren/great grandchildren, respectively: ‘How often do you usually meet and spend time with your child/children (or grandchildren/great grandchildren)?’ (daily; several times a week; a few times a week; a few times a month; a few times a quarter; more seldom or never). This was a general question for grandchildren/great grandchildren, while the question was asked for each individual child. Those who did not have children or grandchildren or did not meet and spend time with any of them at least monthly were classified socially isolated on this indicator (1).

Lack of social contacts with relatives and friends was measured via four items: visiting relatives; having relatives over to visit; visiting friends; and having friends over to visit (no; no, due to the COVID-19 pandemic; yes, sometimes; yes, often). Responding ‘often’ on at least one of these four questions or ‘sometimes’ on at least three questions was classified as not socially isolated on this indicator (0).

The items were summarized into an index with scores ranging from 0 to 3, with higher scores representing higher levels of social isolation. The year 1992 was excluded from the analyses of social isolation due to incomplete information on above mentioned items.

#### Subgroup variables

Sociodemographic variables included age in years [divided into age groups: 77–84 years (1), 85+ years (2)], gender [men (1); women (2)], and education level [‘basic education’ defined as grade school (0); ‘more than basic education’ defined as beyond grade school (1)]. Social variables included living situation [living alone (1); cohabitant (2)].

Health-related variables included mobility and psychological distress. Mobility was measured through the items: ‘Can you walk 100 meters without any difficulties?’ and ‘Can you climb stairs without difficulties?’ [for both: Yes (0); No (1)]. The mobility items were summed into a scale [no mobility limitation (0); mild mobility limitation (1); severe mobility limitation (2)]. Psychological distress was measured by two indicators: self-reported anxiety/worries and depression/deep sadness (for both items: no; yes, slight; yes, severe). Individuals who answered yes to any of the two indicators were coded as having ‘mild or severe psychological distress.’

#### Pandemic-related items

Given that the last wave of data collection was conducted toward the end of the COVID-19 pandemic, questions were asked on how the pandemic had affected social isolation and loneliness. In addition to the response options related to the COVID-19 pandemic regarding contact with friends and relatives, presented above, there were one specific item on the pandemic regarding loneliness and two items regarding contacts with children. The question on loneliness was: ‘To what extent have you been bothered by loneliness during the pandemic compared to previously?’ (more than before; less than before; no difference).

Regarding contacts with children, the following questions were asked: ‘Since the start of the pandemic, has there been any change in how often you meet this child?’ (no, no change; meet more; meet less) and ‘Since the start of the pandemic, has there been any change in how often you talk to this child?’ (no, no change; talk more; talk less). Responses were coded into ‘meeting more’ if they responded that they met more with *any* child regardless of responses regarding other children. Similarly, responses were coded as ‘meeting less’ if participants responded that they met less with *any* child regardless of responses regarding other children. This means that a person who had more contact with one child and less with another child are included in both response groups.

The following questions were asked for grandchildren and great grandchildren: ‘Since the start of the pandemic, has there been any changes in how often you meet your grandchildren and great grandchildren?’ (no change; meet more; meet less) and ‘Since the start of the pandemic, has there been any change in how often you have contact [telephone contact, text message, email, online chat or similar forms of communication] with your grandchildren and great grandchildren?’ (no change, more contact; less contact).

### Analysis

Firstly, descriptive analyses were undertaken to determine the prevalence of loneliness and social isolation status across the years. Thereafter, analyses were done to identify trends in loneliness and social isolation. To analyze change in the distribution of response categories in loneliness and social isolation respectively, we performed ordered logistic regressions. Mean values for the dependent variables (loneliness and social isolation) were calculated for each year. Linear regression analyses were used for tests of statistical significance in trends over time for the full sample and the subgroups. Differences between subgroups in mean loneliness and social isolation were tested via linear regression. Descriptive analyses were conducted for COVID-19 related items to better understand how the pandemic may have influenced the trends in social isolation and loneliness. The association between loneliness and social isolation at different time points was measured via Pearson’s correlation coefficient.

In all analyses including data collected in 1992, 2011, 2014 and 2021, sample weights were applied to adjust for sampling probability. Some individuals have participated in SWEOLD at several waves of data collection, therefore statistical tests were performed with robust standard errors adjusting for clustering over time. Significance levels at *p* < 0.05 are reported as significant. Given the small sample sizes in some subgroups, significance levels between 0.05 and 0.09 are reported as tendencies in the text. Data preparation was done in SPSS v. 29, while data analyses were done in Stata 15.

## Results

### Characteristics of the sample

Across all data collection waves, the majority of the participants were women and the average age was just over 82 years (see Table 1). While persons with basic education constituted 75.7 percent of the sample in 1992, this proportion decreased over the years (*p* < 0.001), particularly between 2014 (51.6%) and 2021 (33.8%). The proportion of participants living alone varied from 45.3 percent (in 2021) to 61.7 percent (in 2004). The mobility improved over the study period (*p* < 0.001), with the lowest proportion of older adults without mobility limitations in 2002 (46.6%) and the highest in 2021 (64.4%). A minority of the participants experienced psychological distress, a proportion that varied between 23.0 percent (in 2021) and 31.9 percent (in 2002).

**Table 1:** Characteristics of the sample.

	1992	2002	2004	2011	2014	2021
	(*n* = 473)	(*n* = 539)	(*n* = 509)	(*n* = 717)	(*n* = 604)	(*n* = 645)
Women %	59.7	57.7	60.3	60.5	58.2	57.2
Mean age (SD)	82.1 (4.1)	82.8 (4.6)	82.6 (4.4)	82.9 (4.8)	82.7 (4.8)	82.4 (4.3)
Age groups %						
77–84 years	74.6	68.5	71.3	67.5	68.4	73.3
85+ years	25.4	31.5	28.7	32.5	31.6	26.8
Basic education^1^ %	75.7	66.2	63.1	53.6	47.0	33.3
Living alone^2^ %	59.3	59.2	61.7	55.3	54.2	45.3
Mobility^3^ %						
No limitation	58.9	46.6	51.1	52.6	62.7	64.4
Mild limitation	17.2	25.3	26.3	22.7	19.0	18.3
Severe limitation	23.9	28.1	22.6	24.6	18.3	17.3
Mild or severe psychological distress^4^ %	25.2	31.9	29.5	32.5	24.0	23.0

1 Item non-response varied between 0 and 20.

2 Item non-response varied between 0 and 5.

3 Item non-response varied between 0 and 3.

4 Item non-response varied between 0 and 12.

### Loneliness and social isolation in older adults over three decades

**Figure 1:**
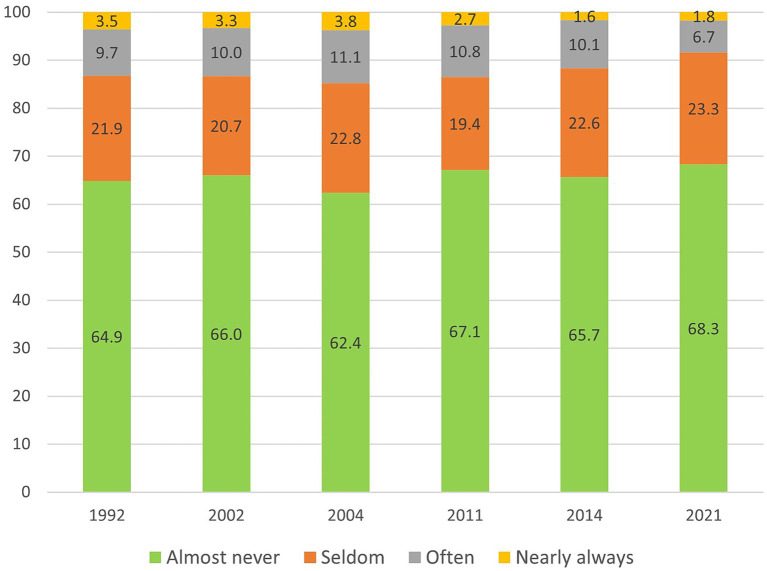
Unadjusted prevalences (%) of loneliness in Sweden, 1992–2021. *n* = 3,439. Weighted data.

The prevalence of loneliness from 1992 to 2021 is presented in Figure 1. Most older adults were seldom or almost never bothered by feelings of loneliness. The proportion of older adults who almost never experienced loneliness varied between 62.4 (in 2004) and 68.3 (in 2021), while around 20 percent seldom experienced loneliness. In total, across all survey years, 12.5 percent were often or almost always bothered by feelings of loneliness. This proportion varied between 14.9 (in 2004) and 8.5 percent (in 2021). The change over time was not statistically significant (OR = 0.99, *p* = 0.145).

**Figure 2:**
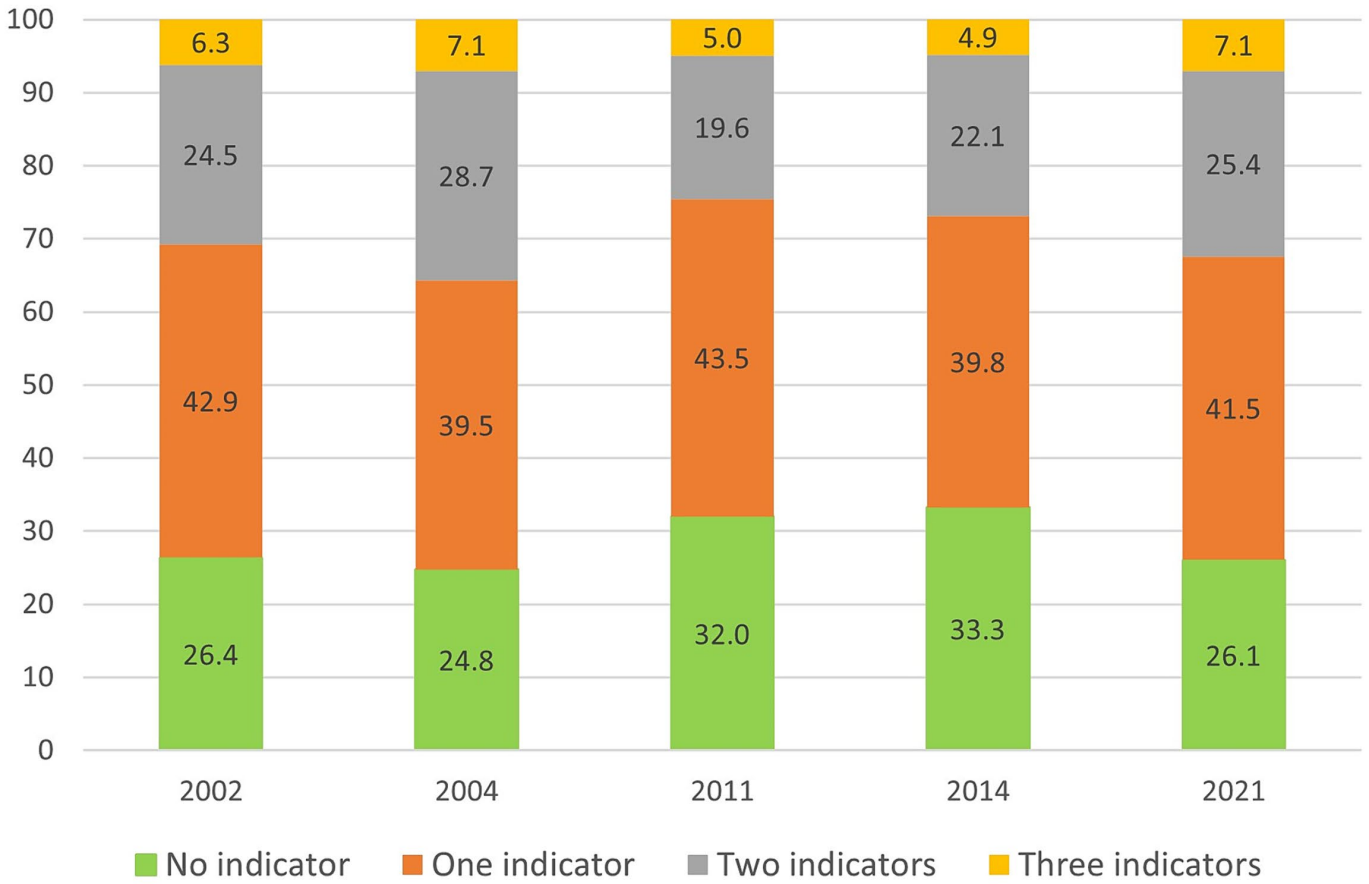
Unadjusted prevalences (%) of up to three indicators of social isolation in Sweden, 2002-2021. *n* = 3,014. Weighted data.

Figure 2 shows that during the period 2002–2021 approximately 6 percent of older adults in Sweden could be categorized as socially isolated, operationalized as receiving the highest score on the social isolation index. The prevalence fluctuated between 4.9 and 7.1 percent but no upward or downward trend was apparent. Another 19.6 to 28.7 percent were isolated on two out of three indicators. Thus, the vast majority of participants were not isolated on any or on one indicator. The change over time was not statistically significant (OR = 0.99, *p* = 0.214).

Given that social isolation includes different forms of social contacts, the trend for each indicator was analyzed separately. Over the study period, there were a decrease in two indicators and an increase in one indicator of social isolation. The proportion of older adults living alone decreased from 59.2 percent in 2002 to 45.3 percent in 2021 (*p* < 0.001). The proportion of participants with low level or no social contacts with children and grandchildren also decreased (*p* = 0.034). In 2002, 27.3 percent had low level/no contacts with children and grandchildren, which increased to 30.3 percent in 2004, followed by a decrease to around 23–24 percent for the following study years. The proportion of older adults with low level of social contacts with relatives and friends increased from 24.4 percent in 2002 to 44.7 percent in 2021 (*p* < 0.001). A large part of this increase occurred between 2014 and 2021, when this indicator of social isolation increased with around 24 percent units.

**Figure 3:**
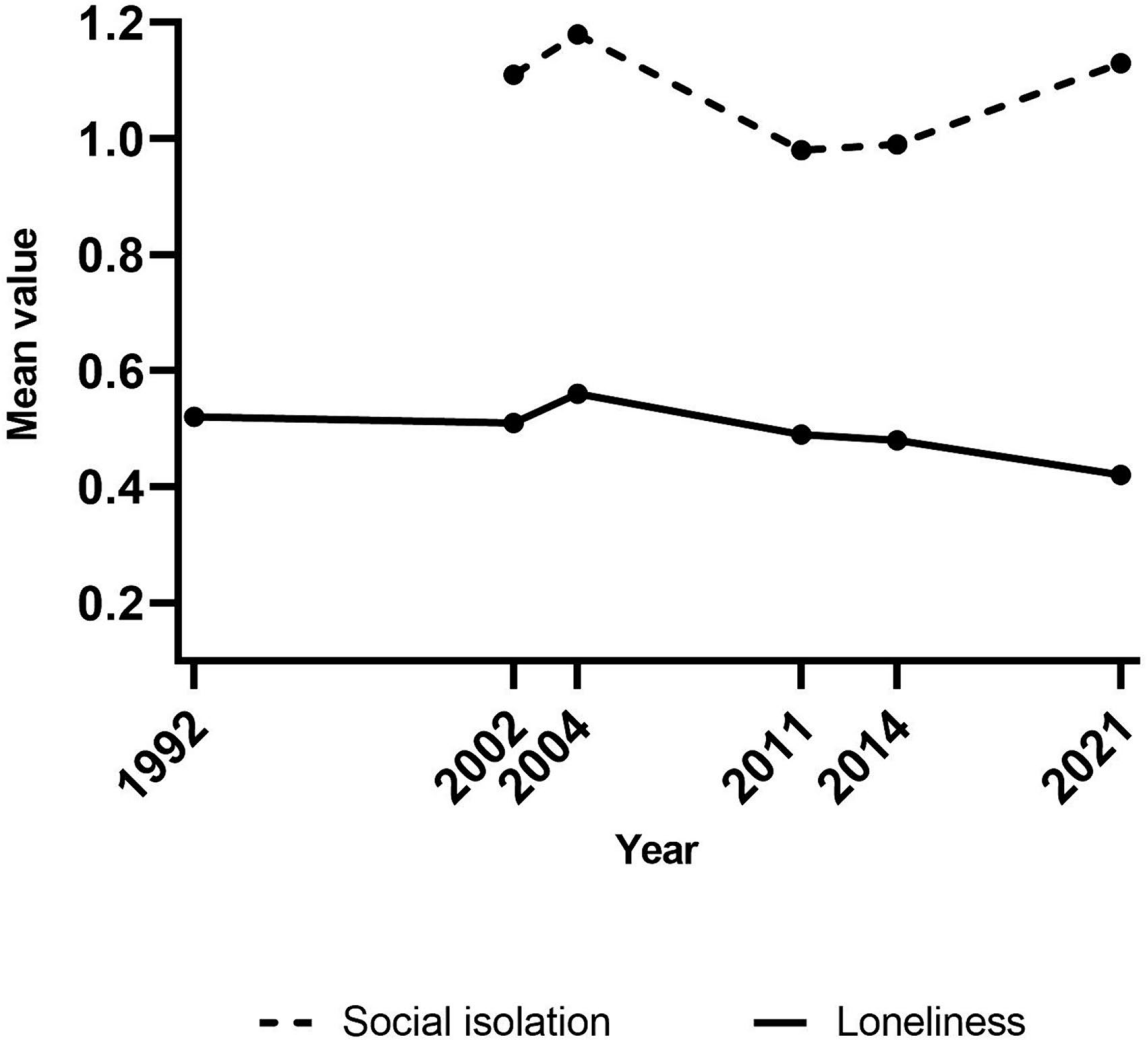
Trends in loneliness (1992–2021, *n* = 3,439) and social isolation (2002–2021, *n* = 3,014) in Sweden. Weighted data. Loneliness and social isolation scores ranged from 0 to 3, with higher scores representing higher levels of loneliness/social isolation.

The mean values of loneliness and social isolation for each survey year are presented in Figure 3. Starting with loneliness, the results show that the mean value was fairly stable over the years but with a decreasing trend (Coeff = −0.003, *p* = 0.047). The mean value for social isolation fluctuated (Coeff = −0.003, *p* = 0.240), with a significant increase since 2011 (Coeff = 0.017, *p* = 0.001). The mean value of social isolation was higher than the mean for loneliness across all years.

### Levels of loneliness and social isolation in subgroups of the population

Levels of loneliness and social isolation were examined in subgroups of the population regarding gender, age, education level, psychological distress, and mobility. For loneliness, the living situation was also examined. Mean levels of loneliness and social isolation are presented in Table 2.

**Table 2:** Total mean levels of loneliness and social isolation in the full sample and subgroups over the study period (weighted).

	Loneliness (*n* = 3,439) M (SD)	Diff. *p* *Β*	Social isolation (*n* = 3,014) M (SD)	Diff. *p* *Β*
Total	0.50 (0.78)		1.07 (0.87)	
Gender		*p* < 0.001		*p* < 0.001
Women	0.58 (0.81)	0.201	1.21 (0.84)	0.331
Men	0.38 (0.72)		0.88 (0.89)	
Age		*p* < 0.001		*p* < 0.001
77–84 years	0.44 (0.74)	0.178	0.96 (0.87)	0.363
85+ years	0.62 (0.86)		1.33 (0.83)	
Education level		*p = 0*.002		*p* = 0.052
Basic education	0.54 (0.81)	−0.106	1.11 (0.87)	−0.081
More than basic education	0.43 (0.73)		1.03 (0.88)	
Living situation		*p* < 0.001		
Living alone	0.74 (0.88)	0.550	–	
Cohabiting	0.19 (0.48)		–	
Mobility		*p* < 0.001		*p* < 0.001
No limitation	0.39 (0.68)		0.93 (0.83)	
Mild limitation	0.52 (0.78)	0.128	1.16 (0.89)	0.441
Severe limitation	0.74 (0.94)	0.350	1.34 (0.87)	0.459
Psychological distress		*p* < 0.001		*p* < 0.001
No distress	0.35 (0.65)	0.525	1.00 (0.87)	0.259
Mild or severe distress	0.87 (0.95)		1.26 (0.85)	

n-size varied across variables due to internal non-response. Loneliness and social isolation scores ranged from 0 to 3, with higher scores representing higher levels of loneliness/social isolation.

Taken together over the study period, the mean level of loneliness was 0.50 for the total sample, 0.58 for women and 0.38 for men (*p* < 0.001). The younger age group had a lower mean loneliness level (0.44) than people aged 85 years or older (0.62; *p* < 0.001). Those with basic education had a higher mean (0.54) than those with more than basic education (0.43; *p = 0*.002). The largest subgroup difference in loneliness was observed between people living alone (0.74) and those cohabiting (0.19; *p* < 0.001), with considerably higher levels of loneliness in those living alone. The mean level of loneliness was higher in the group with mobility limitations (severe: 0.74; mild: 0.52) than among those without such limitations (0.39; *p* < 0.001). Participants with mild or severe psychological distress reported higher levels of loneliness than those without psychological distress, with mean values of 0.87 compared to 0.35 (*p* < 0.001).

The level of social isolation was 1.07 for the entire sample, with higher level in women (1.21 compared to 0.88 for men; *p* < 0.001), in the older age group (77–84 years; 0.96, 85+ years 1.33; *p* < 0.001), in those with lower level of education (basic education: 1.11; more than basic education: 1.03; tendency, *p* = 0.052), in those with mobility limitation (no limitation: 0.93; mild limitation: 1.16; severe limitation: 1.34; *p* < 0.001), and in those with psychological distress (1.26 compared to 1.00 for no psychological distress; *p* < 0.001).

### Trends in loneliness and social isolation for subgroups of the population

**Figure 4:**
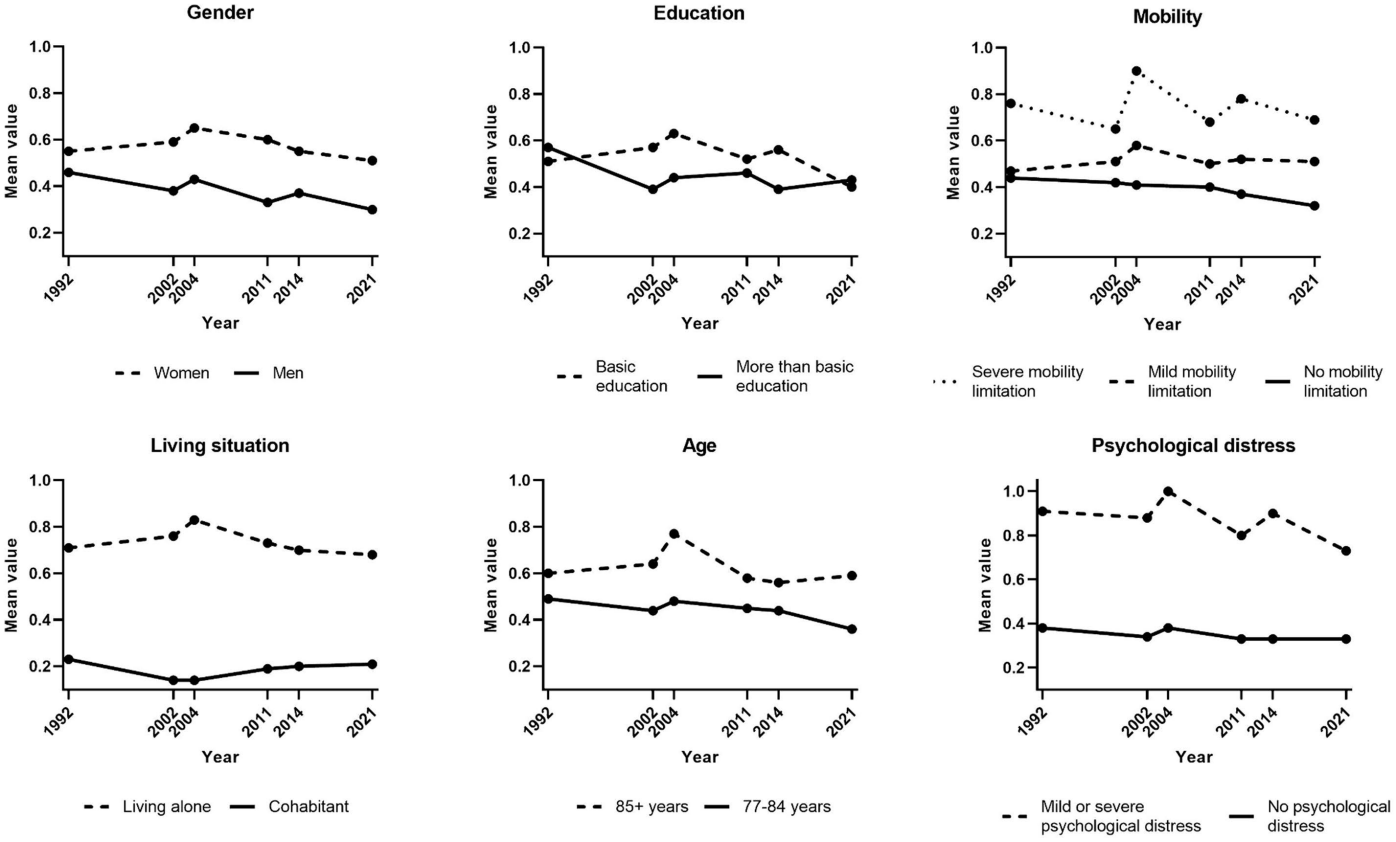
Trends in loneliness (1992–2021) by subgroups. Weighted data. n-size varied across variables due to internal non-response. Loneliness ranged from 0 to 3, with higher scores representing higher levels of loneliness.

Figure 4 presents trends in loneliness for subgroups of the population (for specific results of linear regression analyses, see Supplementary Table S1). This trend was stable for women, whereas there was a tendency toward a decrease in loneliness for men (*p* = 0.056). There was also a tendency of decreasing loneliness in the younger group (*p* = 0.052). The level of loneliness fluctuated over the years for those with mild or severe mobility limitations, while there was a tendency of decreasing loneliness for those without such limitations (*p* = 0.090). No statistically significant trends were found in subgroups regarding education level, living situation or psychological distress.

**Figure 5:**
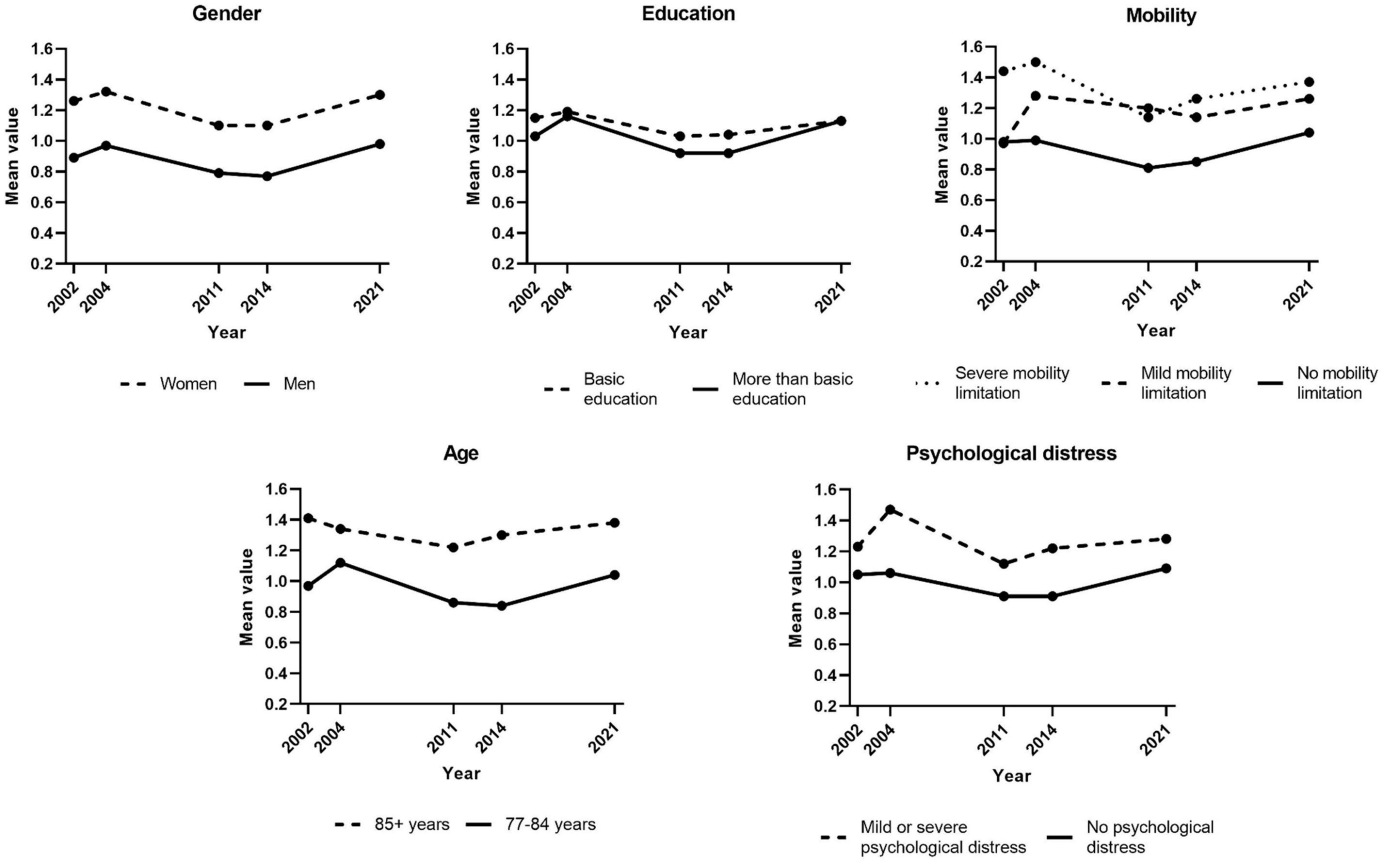
Trends in social isolation (2002–2021) by subgroups. Weighted data. n-size varied across variables due to internal non-response. Social isolation scores ranged from 0 to 3, with with higher scores representing higher levels of social isolation.

Trends in social isolation for population subgroups are presented in Figure 5. Fewer statistically significant changes were found for social isolation. There were no statistically significant changes in social isolation in subgroups based on gender, age, education level, and psychological distress, while there was a tendency of decreasing social isolation in the group experiencing severe mobility limitations (*p* = 0.067).

### Perceived changes due to the COVID-19 pandemic

Changes in loneliness and social isolation over time should be interpreted in relation to restrictions on social interactions during the COVID-19 pandemic. In 2021, 34.2 percent of the respondents reported that they felt lonelier during than before the pandemic and 2.5 percent felt less lonely. Thus, most respondents (63.2%) experienced no change in loneliness during the pandemic compared to the situation before the pandemic.

Respondents also reported that currently low levels of social contacts were due to the pandemic. For example, 38.2 percent did not usually visit friends and 35.0 percent were not usually visited by friends; in both cases more than half of these respondents (22.5 percent units) said that this was due to the pandemic. Similar patterns were observed regarding social contacts with relatives: 45.3 percent did not usually visit relatives and 43.1 percent were not usually visited by relatives, which was due to the pandemic for 18.7 and 19.2 percent units, respectively. Of those who had children, 25.4 percent reported meeting at least one child more often during the pandemic, while 62.0 percent reported meeting at least one child less often. For telephone or chat contact with children, corresponding numbers were 20.5 percent for having more contact and 6.7 percent for less contact. Of those who had grandchildren, 53.7 percent of the respondents met their grandchildren less often during than before the pandemic, while 1.6 percent met them more often. Having contacts with grandchildren was less affected by the pandemic, with the majority (82.4%) reporting no change, 10.6 percent reporting a decrease and 6.9 percent an increase in such contacts.

### Associations between loneliness and social isolation

As presented in Table 3, there was a statistically significant association between loneliness and social isolation each survey year (social isolation not measured in 1992). The strength of the association varied, with the weakest association in 2011 (0.263) and the strongest in 2004 (0.374).

**Table 3:** Associations between loneliness and social isolation for each year^1^ (Pearson Correlation Coefficient).

	*r*	*p*
2002 (*n* = 518)	0.315	<0.001
2004 (*n* = 505)	0.374	<0.001
2011 (*n* = 708)	0.263	<0.001
2014 (*n* = 599)	0.332	<0.001
2021 (*n* = 643)	0.270	<0.001

1 Year 1992 not included, due to unavailability of indicators in the social isolation index.

## Discussion

Based on data from the national survey SWEOLD, this study examined levels and trends in loneliness and social isolation among older adults in Sweden over three decades, assessed variations across subgroups, and determined the strength of association between loneliness and social isolation.

### Levels and trends in loneliness and social isolation

Findings suggest that the majority of people aged 77 years or older were not lonely, with an average of 12.5 percent who were categorized as often or almost always lonely. In line with our previous analyses (18), the present study shows that the prevalence of loneliness has been fairly stable; with the addition of 2021 to the analysis, a slight decrease in the mean score of loneliness was identified. A decrease in loneliness is in line with previous studies with follow-up periods of 20 years or more and starting in the 1990s (22–24), while previous research covering trends during 5–10 years in the 2000s have not identified any significant change in loneliness (19–21). In our study, it is possible that the most recent data collection was influenced by the COVID-19 pandemic. Albeit there have been mixed results, a general increase in loneliness and social isolation in older adults during the COVID-19 pandemic has been noted (17, 25–27). In the present study a third of the participants had felt lonelier during than before the pandemic. It should be noted that data for SWEOLD 2021 was collected between June 2021 and May 2022, that is, toward the end of the pandemic and it is possible that the slight decrease in loneliness reflects a relief of being able to socialize and meet up with friends and family again.

Approximately 5–7 percent of the participants were socially isolated, i.e., they lived alone; did not have children or grandchildren or did not spend time with any of them at least monthly; and had low levels of social contacts with relatives and friends. No significant increase or decrease in social isolation over the whole study period could be identified. A study in the United States has shown no increase in social isolation among people aged 65 years or older between 2003 and 2020 (9). In our study, the higher scores were found in 2004 and 2021, and an increase in social isolation occurred toward the end of the period (since 2011). In part, this increase should be interpreted in the context of the pandemic and the restrictions on social interactions. It is, thus, possible that social isolation may have been more stable or even decreased if the COVID-19 pandemic had not occurred. Other research from Sweden has found that a majority of those being 77 years or older had decreased their in-person contact with family members during the pandemic, and that this decrease was larger for younger old (77–84) than the older old (85+) (35), while there has been an increase in social participation among older in recent decades, disregarding the influence of the pandemic (36). Another observation from our study is that the development of social isolation varied across its different indicators. During the study period, older adults became less isolated on indicators addressing contacts with their closest family, with decreasing proportions of persons living alone and lacking contacts with children and grandchildren. At the same time, it became more common to not have frequent social contacts with relatives and friends.

### Subgroup variations

While only a minority of older adults were lonely and/or isolated, some subgroups of older adults were more exposed to loneliness and social isolation. Subgroup differences were similar for loneliness and social isolation, with higher levels among women, the older age group (85+), persons with basic education, persons with psychological distress, and persons with mild and severe mobility limitations. These findings are generally consistent with previous research [for reviews, see (13, 28–32, 37)]. The higher level of loneliness in women found in this study is also in line with previous research, while the finding of higher levels of social isolation in women than men is more surprising, as men has previously been found to be more socially isolated than women (see reviews cited above). The finding on higher levels of loneliness and social isolation in the older age group has support in previous research on risk factors on loneliness, whereas research on social isolation is more mixed regarding age. Finally, we found loneliness to be more common in those who lived alone; in fact, this is where the greatest subgroup differences in loneliness were found. Again, this echoes previous research findings (see reviews cited above).

Trends in loneliness and social isolation may vary across subgroups and may close or widen the gaps between these groups. This study indicates that the developments of loneliness and social isolation were stable in most subgroups of older adults. Still, there were tendencies toward a decrease in loneliness in men, the younger age group, and those without mobility impairments, i.e., groups that already experienced lower levels of loneliness. Thus, this study suggests that the loneliness gap across subgroups of older adults is widening. Regarding social isolation, a decreasing tendency was only found in people with severe mobility impairments although the general pattern for this group was rather fluctuating. Previous research on subgroup trends in loneliness and social isolation is scarce and the findings are conflicting, so further research is needed in order to draw conclusions on trends of loneliness and social isolation for particular subgroups of the older population.

### Associations between loneliness and social isolation

There was a moderate association between loneliness and social isolation each survey year, which confirms that loneliness and social isolation are related but distinct concepts [see, e.g., (10)]. While cross-sectional analyses cannot say anything about the direction of causality, previous research has shown that aspects of social isolation, such as a limited and decreasing social networks, are longitudinal risk factors for loneliness (13).

### Policy and practice implications

This study disputes the myths of high and increasing levels of loneliness and social isolation in the older population [cf. (38)]. Yet, the general public assumes that the majority of older adults experience loneliness (39). Stereotypes of lonely and isolated older adults are in line with perceptions of older adults as, e.g., incompetent and dependent, and it has been found that ageism has negative effects on loneliness (40). Perceptions of older adults as lonely can also be held by older adults themselves (41). To the extent that loneliness and social isolation are seen as natural and unavoidable elements of aging, this may become a barrier for older adults to develop strategies to change the situation and for practitioners to intervene. In line with this, previous research has shown that the risk of remaining lonely is higher among older adults who view aging as a time of social loss, as they may invest less in new social contacts (42). It is therefore important to avoid stereotypes of aging in, e.g., public debate and media, and instead give a more nuanced view of the heterogeneous older age groups.

Loneliness and social isolation may not be as common as often assumed, but this still leaves a negative impression on everyday life for those affected, and chronic loneliness and severe social isolation also have implications for health and wellbeing. In addition, with aging of the population and extending life spans, the *numbers* of lonely and isolated older adults are increasing. Thus, it is important that policy and practice continue to address these issues with effective and appropriate interventions. Jopling (43) has argued that one of the main challenges for reducing loneliness is to reach persons that experience loneliness. In order to do so, targeting of intervention has to be informed by research on groups vulnerable to loneliness and also social isolation (44). This study contributes with identifying such groups. At the same time, it should be noted that different risk factors for loneliness may be interrelated. For example, marital status and cohabitation reduces the risk of loneliness more in men than in women (45). Therefore, holistic approaches to loneliness are required, including community-based responses to support older adults’ integration in society (46, 47).

### Strengths and limitations

A strength of this study is that it is based on national data, including both older adults living in institutions and in the community. The study includes people with an average age of around 82 years. Given that social interaction develops across the life-course, levels and trends in loneliness and social isolation may be different in younger groups of older adults. SWEOLD has had a high response rate over the years, although this was lower in 2021. Nevertheless, non-response analyses for the 2021 data show that the data is representative of the population in terms of gender and age, whereas the level of education was somewhat higher in the study compared to the national statistics, and attrition analyses show stable levels of loneliness between 2014 and 2021 (48). Another strength is that the data covers a long time-period, enabling analysis of trends in loneliness and social isolation over three decades.

In this study, a direct single item was used to measure loneliness. While this is common [see, e.g., (8, 13)], it could lead to underreporting due to social desirability and bias in interpretation of the item (49), at the same time as direct single items may have high face validity (50). In previous research, social isolation has been operationalized in numerous ways. As no validated scale for social isolation is included in SWEOLD, we constructed a social isolation index in line with the recommendation to include objective and quantitative components of relationships (51). Still, there is a need for national studies generally to include better measurements of loneliness and social isolation.

The mode of data collection varied across data collection waves. In 1992, 2002 and 2011, interviews were primarily done face-to-face, while they were primarily done via telephone in 2004, 2014 and 2021. A recent review suggests that the prevalence of loneliness tends to be higher in face-to-face than telephone interviews (49). In this study, both the highest and lowest prevalence of loneliness were found in years when telephone interviews was the main mode of data collection (2004 and 2021, respectively).

In this study, some results have been reported as tendencies (*p* = 0.05–0.09), as the sample size was small in some subgroups. Thus, the lack of statistically significant results may be due to weak statistical power in some of the analyses. Still, findings reported as tendencies should be interpreted with caution since they can be due to chance.

## Conclusion

This study challenges common perceptions of aging by showing that small proportions of older adults are lonely or experience severe social isolation. Another conclusion is that the prevalences of loneliness and social isolation are not increasing. On the contrary, there is an indication that loneliness may decrease. Nevertheless, loneliness and social isolation are more common in already vulnerable groups, such as persons with basic education, psychological distress and mobility limitations as well as in women and the older age group (85+), while living alone increases the risk of loneliness. These groups should be at the center of attention for efforts to address loneliness and social isolation.

## Data Availability

The data analyzed in this study is subject to the following licenses/restrictions: data are available upon reasonable request, where affiliation to a university is required. Requests to access these datasets should be directed to www.sweold.se.
