# Supplementary material for: National trends in loneliness and social isolation in older adults: an examination of subgroup trends over three decades in Sweden
Source: Front Public Health. 2024 Sep 11;12:1444990. doi: 10.3389/fpubh.2024.1444990 (PMC11422125; doi:10.3389/fpubh.2024.1444990)
Supplement: Supplementary file 1 [file Table_1.pdf]

Lena Dahlberg, Isabelle von Saenger, Mahwish Naseer, Carin Lennartsson, Neda Agahi. National trends in loneliness and social isolation in older adults: An examination of subgroup trends over three decades in Sweden. *Frontiers in Public Health*. DOI 10.3389/fpubh.2024.1444990

Supplementary material

Table S1. Trends in loneliness (year 1992-2021) and social isolation (year 2002-2021) by subgroups assessed through linear regression and presented as B-coefficients with 95% confidence intervals.

Table S1. Trends in loneliness (year 1992-2021) and social isolation (year 2002-2021) by subgroups assessed through linear regression and presented as B-coefficients with 95% confidence intervals.

	Loneliness (n=3439)	95% CI	Social isolation (n=3014)	95% CI
Total	-0.003	-0.007, -0.000	-0.003	-0.009, 0.002
Gender				
Women	-0.002	-0.006, 0.002	-0.005	-0.011, 0.002
Men	-0.005	-0.011, 0.000	-0.0005	-0.009, 0.008
Age groups				
77-84 years	-0.004	-0.008, 0.000	-0.003	-0.009, 0.003
85+ years	-0.003	-0.009, 0.003	-0.002	-0.011, 0.007
Education level				
Basic education	-0.002	-0.006, 0.003	-0.006	-0.013, 0.002
More than basic education	-0.003	-0.009, 0.003	0.001	-0.007, 0.009
Living situation				
Living alone	-0.002	-0.007, 0.003	--	--
Cohabitant	0.0002	-0.003, 0.003	--	--
Mobility				
No limitation	-0.004	-0.008, 0.001	0.0001	-0.006, 0.008
Mild limitation	0.001	-0.006, 0.008	0.008	-0.003, 0.019
Severe limitation	-0.002	-0.009, 0.006	-0.010	-0.021, 0.001
Psychological distress				
No distress	-0.002	-0.005, 0.002	-0.001	-0.007, 0.005
Mild or severe distress	-0.006	-0.014, 0.002	-0.005	-0.015, 0.004

Note 1: n-size varied across subgroups due to internal non-response.
